# Pulse Frequency in Crop Rotations Alters Soil Microbial Community Networks and the Relative Abundance of Fungal Plant Pathogens

**DOI:** 10.3389/fmicb.2021.667394

**Published:** 2021-05-26

**Authors:** Tony Yang, Bianca Evans, Luke D. Bainard

**Affiliations:** ^1^Swift Current Research and Development Centre, Agriculture and Agri-Food Canada, Swift Current, SK, Canada; ^2^Agassiz Research and Development Centre, Agriculture and Agri-Food Canada, Agassiz, BC, Canada

**Keywords:** crop rotation, soil-borne disease, pulse frequency, fungal guilds, network analysis

## Abstract

Including pulse crops in cereal-based cropping systems has become a widely accepted and useful agronomic practice to increase crop diversification and biologically fixed nitrogen in agroecosystems. However, there is a lack of knowledge regarding how the intensification of pulses in crop rotations influence soil microbial communities. In this study, we used an amplicon sequencing approach to examine the bulk and rhizosphere soil bacterial and fungal communities from the wheat (*Triticum aestivum* L.) phase (final year of 4 years rotations) of a long-term pulse intensification field trial in the semi-arid region of the Canadian Prairies. Our results revealed pulse frequency had a minimal impact on microbial α-diversity, but caused a significant shift in the composition of the fungal (rhizosphere and bulk soil) and bacterial (bulk soil) communities. This effect was the most pronounced in the Ascomycete and Bacteroidete communities. Increasing pulse frequency also promoted a higher proportion of fungal pathotrophs in the bulk soil, particularly those putatively identified as plant pathogens. The network analysis revealed that rotations with higher pulse frequency promoted increased competition within the soil microbial networks in the rhizosphere and bulk soil. However, we also detected more negative interactions among the dominant pathotrophic taxa with increased pulse frequency, suggesting higher soil-borne disease potential. These findings highlight the potential drawbacks and reduced sustainability of increasing pulse frequency in crop rotations in semiarid environments.

## Introduction

Among agronomic practices used in annual cropping systems, crop rotation is one of the most common management tools used to enhance soil nutrient and water availability, control weeds and pests, and improve the ecological and economic sustainability of cropping systems (Schönhart et al., 2011; Barbieri et al., 2017). Considering the unique capability of legumes to fix atmospheric nitrogen via biological nitrogen fixation (BNF), legume-based rotations have demonstrated many advantages for enhancing the sustainability of agriculture worldwide. As a result, the land base planted to legume crops has gradually increased globally over the past 50 years (Foyer et al., 2016).

This trend has been particularly evident in the Northern Great Plains for pulse crops such as field pea (*Pisum sativum* L.), lentil (*Lens culinaris Medik*.), and chickpea (*Cicer arietinum* L.), which are common grain legume crops that have been used to diversify cereal and oilseed cropping systems (Khakbazan et al., 2020). The globally cultivated area for pea, lentil and chickpea is around 8.1, 6.6, and 14.6 Mha, respectively, which provide about 0.74, 0.36, and 0.66 Tg of annual fixed nitrogen, respectively (Jensen et al., 2020). Including pulses in crop rotations has been documented to provide many economic and ecological benefits including increased soil organic carbon (Liu k. et al., 2020) and nitrogen availability (Jensen et al., 2020), reduced disease pressure (Kirkegaard et al., 2008), increased yield of subsequent crops in rotations (Reckling et al., 2020; Zhao et al., 2020) and overall system productivity (Gan et al., 2015), and improved soil health (Lal, 2017). However, due to the application of synthetic fertilizers and pesticides as well as economic drivers of crop selection, diversification of many crop rotation designs in practice are overly simplified, and improper rotation designs in modern agriculture jeopardize the eco-functionality of agroecosystems and endanger soil biodiversity (Barbieri et al., 2017). It is essential to select the right type of pulse crops for the rotation design and ecosystem, and incorporate them at an appropriate frequency to achieve the potential ecological and economic benefits linked to these crops. However, this type of information is still very limited, particularly from long-term field studies.

In particular, our understanding of how pulse based mid- to long-term rotations and high pulse frequency rotations influence soil microbial community dynamics remains limited. Early evidence from short term studies (<4 years) has shown that pulse frequency in annual crop rotations is an important factor driving shifts in the composition, diversity and functional guilds of soil and root-associated microbial communities (Bainard et al., 2017; Navarro-Borrell et al., 2017; Hamel et al., 2018; Niu et al., 2018). There is evidence that plant species can have selective effects on rhizosphere microbial communities. As a result, enriched pulse frequency in annual crop rotations will likely lead to a gradual enrichment of host specific effects in soil, which can further alter soil microbial communities.

In this study, we used a long term (8 years) crop rotation study in the semiarid region of the Canadian Prairies to explore the impacts of pulse frequency on the diversity, composition and interactions of soil microbial communities. We hypothesized that increasing the pulse frequency in crop rotations will alter the diversity, composition, and network interactions of soil microbial communities.

## Materials and Methods

### Site Description and Experimental Design

A long-term field experiment was established in 2010 with a 4 years crop rotation design located at the Swift Current Research and Development Centre was utilized for this study. The experiment included low (one-pulse phase), medium (two-pulse phases) and high (three-pulse phases) pulse frequency rotations in the long-term rotation design. For this study we selected the following treatments: (i) low pulse frequency rotations, Pea-Wheat-Wheat-Wheat (PWWW) and Chickpea-Wheat-Wheat-Wheat (CWWW); (ii) medium pulse frequency rotations, Pea-Wheat-Pea-Wheat (PWPW) and Chickpea-Wheat-Chickpea-Wheat (CWCW); and (iii) high pulse frequency rotations, Pea-Pea-Pea-Wheat (PPPW), Chickpea-Chickpea-Chickpea-Wheat (CCCW) and Pea-Lentil-Chickpea-Wheat (PLCW) rotations. The first cycle of the rotations was completed in 2013 and repeated again starting in 2014. Due to the development of serious root diseases issues associated with growing chickpea in the same plots for three continuous years during the first cycle, mustard was grown during the second year of the CCCW rotation during the second cycle (i.e., Chickpea-Mustard-Chickpea-Wheat) to limit disease issues. Each treatment was planted into a 4 × 12 m plot and replicated four times in a randomized complete block design. This study was conducted during the 2017 growing season, which was the fourth year of the rotations during the second cycle and all rotations were seeded to wheat (*Triticum aestivum* L.).

All plots received glyphosate spray at 0.45 kg/ha as a pre-seeding burn-off treatment and Trifluralin 10G at 8.5 kg/ha prior to seeding (except for wheat plots) to eliminate weed effects in each year. At the beginning of each rotation cycle (2010 and 2014) all wheat plots received a total of 80 Kg/ha actual N using 46-0-0 mineral fertilizer, and pulse crop plots received 22 kg/ha of actual P using 11-51-0 mineral fertilizer. All pulse plots also received inoculation prior to seeding with a granular rhizobium inoculant (TeamTag^®^, 4kg ha^-1^) annually. All plots were under no-till management practices and were planted with a no-till plot seeder.

### Sampling and Chemical Analysis

Soil sampling was conducted at the soft dough stage to assess the soil microbial community and soil chemical properties. Four soil cores (2.5 cm diameter) were randomly collected in each plot to a depth of 15 cm, pooled together and homogenized in a large Ziploc bag to form one composite soil sample per plot. At the same time, four wheat plants were carefully excavated from the same sampling locations as the bulk soil cores in each plot. The loose soil was shaken from the roots and the remaining soil adhering to the roots was carefully brushed off, pooled together and homogenized as one rhizosphere soil sample per plot. There were a total of 28 bulk soil and 28 rhizosphere soil samples collected for this study. Sub-samples were immediately taken from the composite bulk and rhizosphere soil samples and flash frozen in a liquid nitrogen cryo-shipper for molecular analyses. The remaining soil was stored in a cooler with ice for transportation and then stored at 4°C for up to 48 h until further processing. Soil samples were sieved through a 2 mm sieve, and a subsample was used to determine soil moisture content (gravimetric). The remaining soil was air-dried and ground for chemical analyses.

For all soil samples, soil organic carbon and total carbon were determined using the dry combustion method (after acidification with HCl) using an Elementar vario MICRO cube elemental analyzer (Schumacher, 2002). Soil nitrate nitrogen (N), phosphate phosphorus (P) and potassium (K) were determined using sodium bicarbonate extractions followed by colorimetric analysis using a Technicon Autoanalyzer (Harm et al., 1973; Gentry and Willis, 1988). Soil sulfate sulfur (S) was determined using calcium chloride extractions followed by colorimetric analysis using a Technicon Autoanalyzer (Harm et al., 1973). Soil pH and EC were measured in water saturation paste (Habekost et al., 2008) and paste extracts (Miller and Curtin, 2006). Soil moisture was measured using the gravimetric method.

### DNA Extraction, Amplicon Sequencing, and Bioinformatics

Genomic DNA was extracted from 0.25 g of soil in duplicate for each bulk and rhizosphere soil sample using the DNeasy PowerSoil kit (Qiagen) using the QIAcube automated nucleic acid extraction system (Qiagen). DNA was quantified using a Qubit dsDNA BR Assay Kit (Thermo Fisher Scientific, Waltham, MA) and shipped on dry ice to the Genome Quebec Innovation Center (Montreal, Canada) for amplicon library preparation and Illumina MiSeq sequencing. The bacterial 16S rRNA genes were sequenced using primers 515-F and 806-R (Caporaso et al., 2012) and fungal ITS1 region was sequenced using primers ITS1F and 58A2R (Martin and Rygiewicz, 2005). For a full description of the amplicon library preparation and Illumina Miseq sequencing see Delavaux et al. (2020). The raw amplicon sequencing datasets are available in the NCBI Sequence Read Archive under BioProject ID PRJNA694704, PRJNA694839, PRJNA694691, PRJNA694475, PRJNA717779, and PRJNA694448. Raw reads were processed (assembled, trimmed, quality filtered, dereplicated, and clustered) using the UPARSE pipeline of USEARCH v.9 (Edgar, 2013). Sequences were clustered into operational taxonomic units (OTU) based on 97% similarity. Taxonomic identity was assigned with a confidence threshold of 80% using the RDP classifier (Wang et al., 2007) and 16S rRNA training set (version 16) for bacteria and ITS UNITE database for fungi (Kõljalg et al., 2013). FUNGuild was used to parse the fungal community datasets by functional ecological guild (Nguyen et al., 2016). See Bainard et al. (2020) for a full description of the bioinformatics.

### Statistical Analysis

All statistical analyses were performed using R packages (version 3.5.1) vegan, breakaway, ecodist and ggplot2 (R Foundation for Statistical Computing, Vienna, Austria). To determine the effect of pulse frequency on α-diversity we estimated the total OTU richness using the package breakaway (Willis and Bunge, 2015). One-way analysis of variance (ANOVA) was used to test the effect of pulse frequency on the soil properties and OTU richness. *Post hoc* comparisons of means were completed using Tukey’s HSD procedure at a 5% level of probability. Permutational multivariate analysis of variance (permanova) was used to test the effect of pulse frequency on the wheat rhizosphere and bulk soil microbial communities. Sequence read variability was normalized among samples by randomly resampling to the lowest number of reads for each dataset (i.e., bacteria = 19,682 reads/sample; fungi = 21,468/sample). To eliminate influences of low abundance OTUs which are in many cases artifacts generated through the sequencing process and better compare treatment effects, unclassified OTUs and rare OTUs (OTUs shown in less than 3 soil samples and OTUs with less than 10 reads in total) were removed from downstream analysis. Network analysis was constructed for soil microbial communities related to different pulse frequencies based on their OTU relative abundances, and was performed using the Molecular Ecological Network Analyses (MENA) pipeline: http://ieg4.rccc.ou.edu/mena (Deng et al., 2012) and visualized by Cytoscape 3.6.1. Module-EigenGene network analysis was applied to examine interactions of the soil microbial community with environmental factors. The connectivity of each node was determined based on its within-module connectivity (Zi) and among-module connectivity (Pi), and the node topologies were separated by Zi and Pi with threshold of 2.5 and 0.62 (Guimerà and Nunes Amaral, 2005; Olesen et al., 2007). OTUs with low Zi and low Pi values were considered as specialists (peripherals) which typically only had links within their own modules. OTUs with low Zi and high Pi values (connectors), or high Zi and low Pi values (module hubs) were considered as generalists. This included either module hubs (actively linked with other OTUs within their own module) or connectors (linking with OTUs from other modules). OTUs with high Zi and Pi values were considered as super generalists (network hubs) which act as both connectors among several modules and module hubs within their own modules.

## Results

### Soil Properties and Wheat Growth

Pulse frequency in the rotations had a minimal impact on the bulk and rhizosphere soil properties. Total C and organic C in rhizosphere soil was significantly higher in rotations with medium pulse frequency compared to rotations with low and high pulse frequency, and soil moisture was highest in rotations with low pulse frequency (Table 1). In the bulk soil, rotations with a medium pulse frequency had a significantly higher soil S and P content compared to the high pulse frequency rotations. All other soil properties in the bulk and rhizosphere soil were unaffected by pulse frequency. Pulse frequency also impacted wheat growth. Tiller number and dry biomass of wheat were significantly affected by pulse frequency, with rotations with medium pulse frequency exhibiting the highest biomass production. However, wheat yield, height or seeds per plant did not significantly differ among the rotations with different pulse frequencies (Supplementary Table 1).

**TABLE 1:** ANOVA results of the effect of pulse crop frequency (low, medium, and high) on the wheat rhizosphere and bulk soil properties.

**Sample type**	**Pulse frequency**	**S (mg kg^–1^)**	**K (mg kg^–1^)**	**N (mg kg^–1^)**	**P (mg kg^–1^)**	**TC (%)**	**OC (%)**	**TN (%)**	**pH**	**EC (dS m^–1^)**	**Moisture (%)**
Rhizosphere	Low	1.49 ± 0.10	253.48 ± 8.96	3.73 ± 0.60	27.94 ± 0.95	1.46 ± 0.04^b^	1.40 ± 0.03^b^	0.14 ± 0.003	6.34 ± 0.09	0.28 ± 0.02	12.76 ± 0.20^a^
	Medium	2.21 ± 0.23	267.15 ± 13.69	5.37 ± 0.68	26.85 ± 0.95	1.53 ± 0.02^a^	1.46 ± 0.02^a^	0.15 ± 0.002	6.48 ± 0.14	0.40 ± 0.05	11.88 ± 0.60^b^
	High	1.82 ± 0.10	238.86 ± 9.79	4.88 ± 0.53	31.15 ± 1.28	1.47 ± 0.04^b^	1.40 ± 0.04^b^	0.14 ± 0.003	6.60 ± 0.10	0.32 ± 0.03	11.43 ± 0.21^b^
	*P*-value	0.224	0.158	0.292	0.738	0.009	0.013	0.215	0.961	0.678	0.017
Bulk soil	Low	2.56 ± 0.22^ab^	270.45 ± 14.17	6.44 ± 1.49	64.86 ± 3.45^ab^	1.66 ± 0.04	1.55 ± 0.03	0.16 ± 0.002	6.29 ± 0.07	0.35 ± 0.04	10.10 ± 0.27
	Medium	2.87 ± 0.18^a^	273.78 ± 12.10	5.42 ± 0.99	68.01 ± 2.17^a^	1.55 ± 0.03	1.44 ± 0.03	0.15 ± 0.003	6.32 ± 0.10	0.36 ± 0.03	9.22 ± 0.16
	High	2.42 ± 0.15^b^	242.25 ± 10.68	8.19 ± 1.18	63.37 ± 2.29^b^	1.51 ± 0.03	1.45 ± 0.03	0.15 ± 0.003	6.29 ± 0.11	0.32 ± 0.02	9.41 ± 0.21
	*P*-value	0.01	0.207	0.161	0.021	0.365	0.46	0.246	0.235	0.084	0.108

*Means (±standard error) for each sample type in the same column with different letters are significantly different (P < 0.05).*

### Soil Microbial Diversity

**FIGURE 1:**
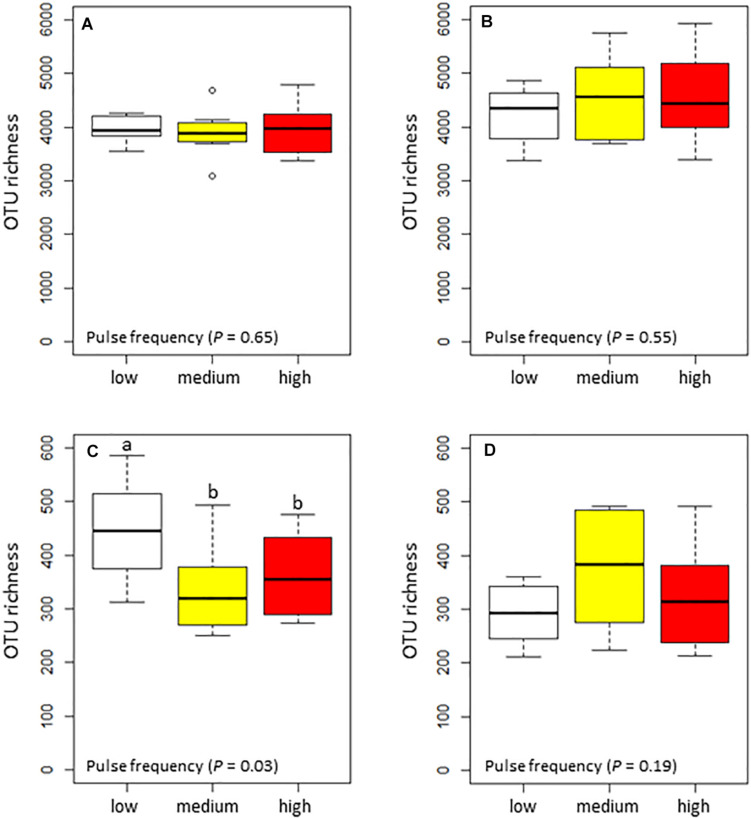
Box plots of the total estimated richness for the bacterial community in the **(A)** wheat rhizosphere and **(B)** bulk soil, and fungal community in the **(C)** wheat rhizosphere and **(D)** bulk soil as influenced by pulse frequency in the crop rotations.

Pulse frequency did not significantly affect the α–diversity (estimated total richness) of the bacterial community in the rhizosphere or bulk soil (Figures 1A,B). However, pulse frequency did significantly affect the estimated total fungal richness in the rhizosphere (highest under low pulse frequency), but not in the bulk soil (Figures 1C,D). Among the fungal guilds, pathotrophs were the only group that were significantly affected by pulse frequency (Table 2). In the rhizosphere, rotations with medium pulse frequency had the highest proportion of fungal pathotrophs. In the bulk soil, rotations with medium and high frequency had the highest proportion of fungal pathotrophs. Of the fungal pathotrophs putatively assigned as plant pathogens in the bulk soil, the rotations with medium and high pulse frequency had nearly twice the proportion of reads compared to the rotations with low pulse frequency.

**TABLE 2:** ANOVA results of the effect of pulse crop frequency (low, medium, and high) on the proportion (percentage) of reads assigned to fungal guilds in the wheat rhizosphere and bulk soil.

**Sample type**	**Pulse frequency**	**Pathotrophs (%)**	**Saprotrophs (%)**	**Symbiotrophs (%)**	**Undefined (%)**	**Plant pathogens (%)**
Rhizosphere	Low	21.0 ± 1.2^b^	52.2 ± 2.6	0.7 ± 0.2	11.3 ± 1.6	14.8 ± 0.8
	Medium	26.5 ± 1.1^a^	43.4 ± 2.2	1.1 ± 0.4	12.0 ± 2.6	17.1 ± 1.7
	High	21.2 ± 2.1^b^	44.5 ± 4.2	0.3 ± 0.2	16.4 ± 7.6	17.5 ± 1.7
	*P*-value	0.035	0.269	0.128	0.722	0.248
Bulk soil	Low	18.6 ± 2.4^b^	48.2 ± 4.2	1.3 ± 0.8	20.0 ± 4.4	11.9 ± 1.6^b^
	Medium	29.4 ± 2.0^a^	38.6 ± 2.9	0.3 ± 0.2	11.2 ± 1.0	20.5 ± 1.6^a^
	High	26.4 ± 1.7^a^	42.3 ± 3.0	0.0 ± 0.0	10.3 ± 1.3	21.0 ± 1.2^a^
	*P*-value	0.001	0.427	0.142	0.070	<0.001

*Means (± standard error) for each sample type in the same column with different letters are significantly different (P < 0.05).*

The permanova indicated that pulse frequency had a significant effect on the fungal community in both the rhizosphere and bulk soil (Table 3). This appears to be primarily linked to shifts in the taxa belonging to the Ascomycota in bulk soil, and taxa belonging to the Ascomycota and Mortierellomycota in rhizosphere soil. The principle coordinate analyses (PCoA) revealed that the fungal community from rotations with low pulse frequency were distinct and showed some separation compared to those from medium and high pulse frequency in both the rhizosphere and bulk soil (Supplementary Figure 1). This trend is particularly evident for the Ascomycete community in both the rhizosphere and bulk soil. Pulse frequency did not have a significant effect on the bacterial community in either the bulk or rhizosphere soil. However, pulse frequency did significantly influence the rhizosphere Firmicutes community, and bulk soil bacterial communities belonging to the Actinobacteria, Armatimonadetes, Bacteroidetes, candidate division WPS-1, Gemmatimonadetes, and Proteobacteria (Table 3 and Supplementary Figure 2). The strongest effect was observed on the Bacteroidetes community in the bulk soil, with the PCoA showing a distinct separation between samples taken from rotations with low pulse frequency and those from medium and high pulse frequency (Supplementary Figure 2).

**TABLE 3:** Permanova results of the effect of pulse crop frequency (low, medium, and high) on the wheat rhizosphere and bulk soil fungal and bacterial community composition.

	**Rhizosphere soil**			**Bulk soil**		
	**F**	**R^2^**	***P***	**F**	**R^2^**	***P***
***Fungi***						
Total	1.3	0.093	0.05	1.845	0.129	0.001
Ascomycota	1.485	0.106	0.014	2.106	0.144	<0.001
Basidiomycota	0.918	0.068	0.627	1.074	0.079	0.33
Mortierellomycota	0.889	0.066	0.541	1.733	0.122	0.049
***Bacteria***						
Total	1.371	0.099	0.165	1.632	0.115	0.06
Acidobacteria	1.533	0.109	0.146	1.393	0.1	0.159
Actinobacteria	1.296	0.093	0.208	1.89	0.131	0.031
Armatimonadetes	1.217	0.089	0.234	1.831	0.128	0.031
Bacteroidetes	1.236	0.09	0.176	2.502	0.167	<0.001
Candidate division WPS-1	1.127	0.083	0.318	1.6	0.113	0.042
Chloroflexi	0.898	0.067	0.527	1.316	0.095	0.186
Firmicutes	2.382	0.16	0.037	0.359	0.028	0.904
Gemmatimonadetes	1.305	0.095	0.195	2.155	0.147	0.025
Nitrospirae	2.252	0.153	0.068	1.955	0.135	0.054
Planctomycetes	1.004	0.074	0.397	1.486	0.106	0.06
Proteobacteria	1.482	0.106	0.109	1.986	0.137	0.013
Thaumarchaeota	0.959	0.072	0.454	1.022	0.076	0.417
Verrucomicrobia	1.106	0.081	0.314	1.269	0.092	0.226

### Soil Microbial Community Networks

**FIGURE 2:**
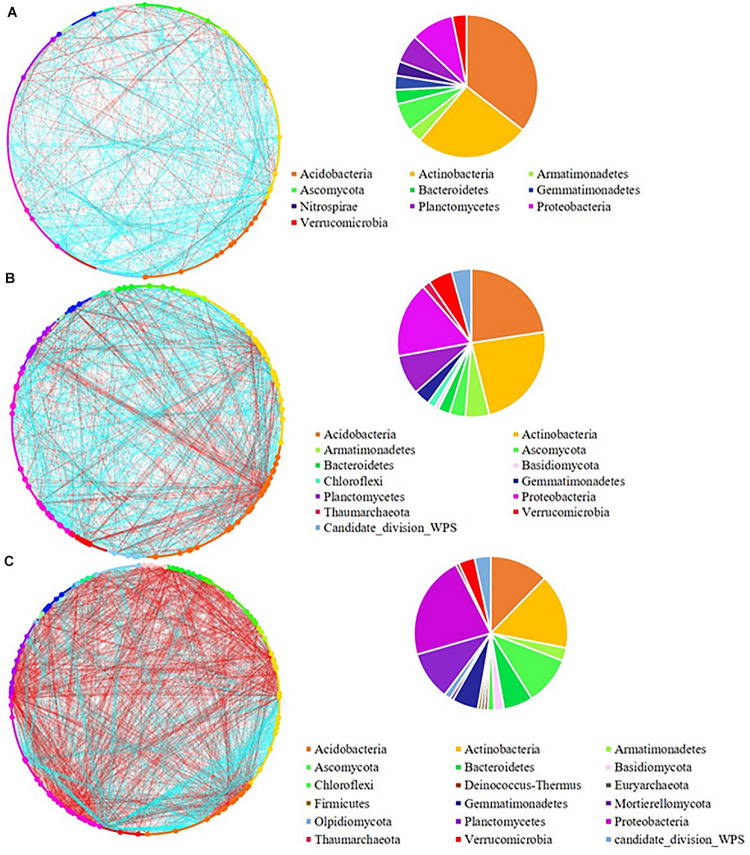
Network analysis of **(A)** low; **(B)** medium, and **(C)** high pulse frequency influenced microbial community and dominant microbial OTUs (larger dots in network, OTUs with node degree larger than 10) in wheat rhizosphere soil. Blue lines between any connected two nodes indicates a positive relationship and red lines indicate a negative relationship. The pie graph associated with each network shows the composition of the dominant microbial OTUs (nodes) in the community.

**FIGURE 3:**
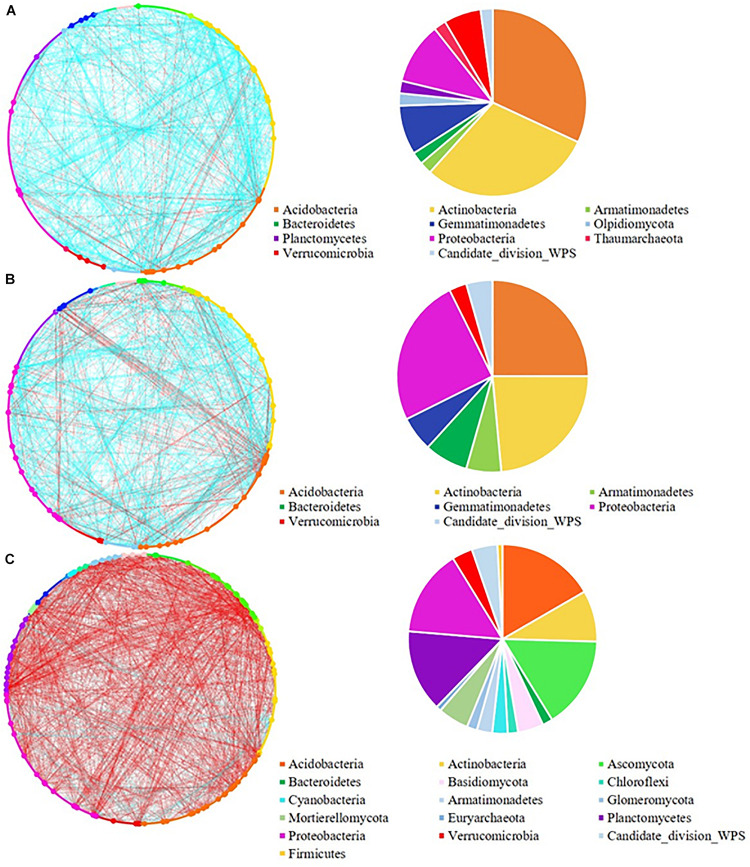
Network analysis of **(A)** low; **(B)** medium, and **(C)** high pulse frequency influenced microbial community and dominant microbial OTUs (larger dots in network, OTUs with node degree larger than 10) in bulk soil. Blue lines between any connected two nodes indicates a positive relationship and red lines indicate a negative relationship. The pie graph associated with each network shows the composition of the dominant microbial OTUs (nodes) in the community.

The network analysis revealed that increasing the pulse frequency in crop rotations modified the interactions of the soil microbial community (Table 4). The top three dominant bacterial phyla (Acidobacteria, Actinobacteria, and Proteobacteria) influenced the structure and interconnectedness of the soil microbial community. The majority of the nodes with high degree in the network analysis in both rhizosphere and bulk soil were linked to these three dominant phyla (Acidobacteria > Actinobacteria > Proteobacteria), and accounted for approximately 50% of the identified dominant nodes (Figures 2, 3). In both rhizosphere and bulk soil, rotations with high pulse frequency had microbial communities with stronger interactions that included a smaller number of nodes, higher number of edges, shorter average path distance, more connectedness, higher betweenness centrality, less modules and more dominant nodes (Table 4). These results suggest that high levels of pulse frequency in crop rotations can enhance the soil microbial community interactions.

**TABLE 4:** Network topological properties of the microbial community identified in the wheat rhizosphere and bulk soil samples in rotations with low, medium or high pulse frequency.

		**Nodes**	**Edges**	***R*^2^ of power law**	**Average path distance**	**Average clustering coefficient**	**Connectedness**	**Betweenness centrality**	**Module number**	**Nodes with degree > 10**
Rhizosphere	Low	1,010	1,491	0.889	7.490	0.104	0.537	0.103	26	31
	Medium	816	2,187	0.879	4.746	0.105	0.572	0.060	19	115
	High	787	2,983	0.950	4.355	0.060	0.814	0.110	8	146
Bulk soil	Low	897	1,618	0.882	7.377	0.135	0.476	0.066	30	47
	Medium	843	1,634	0.921	5.669	0.120	0.528	0.088	18	69
	High	831	2,110	0.916	4.327	0.068	0.660	0.101	8	114

Increasing pulse frequency also increased phylogenetic diversity of dominant nodes in the rhizosphere (Figure 2 and Supplementary Figure 3), especially taxa that belonged to the Basidiomycota, Chloroflexi, Thaumarchaeota and candidate division WPS-1. The number of nodes from Ascomycota, Gemmatimonadetes, and Proteobacteria were increased while the number of nodes from Acidobacteria and Actinobacteria were decreased with increased pulse frequency. We also observed a higher number of negative interactions between nodes under high pulse frequency than under low pulse frequency, which may be linked to higher competition between soil microorganisms for limited nutrients and environmental resources, or accumulation of pulse specific pathogens.

In the bulk soil, high pulse frequency led to an increased total phylogenetic diversity of dominant nodes along with a shift in composition compared with low and medium pulse frequency rotations (Figure 3 and Supplementary Figure 4). For example, dominant nodes from Acidobacteria, Actinobacteria, and Gemmatimonadetes were reduced and dominant nodes from Ascomycota, Basidiomycota, Mortierellomycota, Planctomycetes and candidate division WPS-1 were increased in rotations with high pulse frequency. The latter group accounted for approximately half of the total dominant nodes and the majority of the negative interactions with other nodes in the bulk soil microbial community.

### Soil Microbial Community Response to Environmental Factors

Module-EigenGene network analysis grouped soil microorganisms into different modules based on their interactions with environmental factors (Supplementary Figure 5). In both the rhizosphere and bulk soil, module numbers decreased with an increase in pulse frequency, indicating that rotations with higher frequency of pulses can strength interactions within the microbial community. However, the microbial community in bulk soil and rhizosphere soil reacted to the pulse frequency differently.

In the rhizosphere, S, pH and EC had the strongest influence on the soil microbial community (Supplementary Figures 5A–C and Supplementary Table 1). This was particularly evident for the bacterial community under low to medium pulse frequency rotations. The modules that contained the keystone OTUs (black framed modules, Supplementary Figures 5A–C) were more sensitive to EC and pH under low to medium pulse frequency rotations, but more sensitive to TC and TN under high pulse frequency. This indicated that the influence of pulse crops on microbial keystone taxa in the rhizosphere is co-dependent on the pulse frequency and environmental factors.

In the bulk soil, S, N, and K had the strongest influence on the soil microbial community in low pulse frequency rotations (Supplementary Figure 5D), while N had the strongest influence in medium pulse frequency rotations (Supplementary Figure 5E). Under high pulse frequency rotations, EC had the strongest effect (Supplementary Figure 5F), particularly for the bacterial community (Supplementary Table 1). For the modules that include most of the keystone taxa, they had a negative response to S under low pulse frequency, and strong response to N (either negative or positive, depending on the module) under medium pulse frequency, and none of these environmental factors significantly affected these modules under high pulse frequency. These results suggest that in bulk soil, rotations with a high frequency of pulse crops could develop a relatively stable soil microbial community network that is not as responsive to changing environmental conditions.

### Impact of Pulse Frequency on Keystone Taxa

**FIGURE 4:**
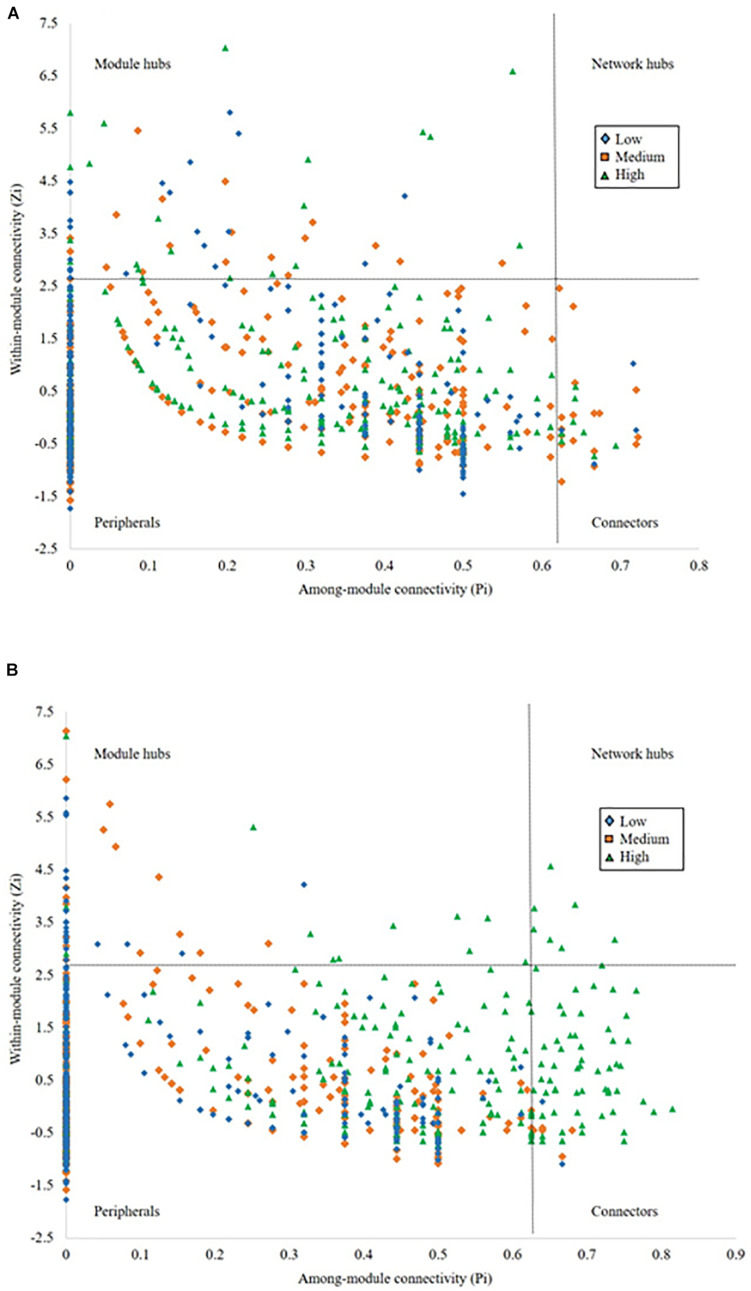
The network nodes of **(A)** rhizosphere and **(B)** bulk soil were separated by within-module connectivity (Zi) and among-module connectivity (Pi).

To evaluate the topological role of identified nodes in the network, we used within-module connectivity (Zi) and among-module connectivity (Pi) values to separate each network into four ecological modules (Figure 4). From the rhizosphere soil (Figure 4A), we identified 22 module hubs and 4 connectors in low pulse frequency, 20 module hubs and 17 connectors in medium pulse frequency, 25 module hubs and 11 connectors in high pulse frequency. From bulk soil (Figure 4B), we identified 23 module hubs and 2 connectors in low pulse frequency, 18 module hubs and 8 connectors in medium pulse frequency, 14 module hubs, 74 connectors, and 9 network hubs in high pulse frequency. The higher number of connectors under high pulse frequency indicated that soil microbial taxa were more active within their own modules under low pulse frequency, but tended to build up interactions with OTUs located at different modules under high pulse frequency. These results suggest that higher levels of pulse frequency in crop rotations can enhance the interactions of soil microbial taxa in different subgroups which may carryout different biological and ecological functions.

## Discussion

Despite the important role that crop rotations play in sustainable agriculture (Drinkwater et al., 1998; Foyer et al., 2016), there is still a lack of systematic analysis on how pulse crops influence soil microbial community dynamics (Hamel et al., 2018). The results from our study provide insight into how pulse frequency in crop rotations impacts the soil microbial community, their diversity, interactions, potential eco-functions and relationship with environmental factors.

Soil bacterial diversity did not significantly change in response to increased pulse frequency in the crop rotations. Similar results were observed during the first rotation cycle at the same field trial and phase of the rotation (Hamel et al., 2018), but these results were based on the bacterial community colonizing the wheat roots rather than the rhizosphere or bulk soil. A recent meta-analysis also found that although some rotations can have either a negative or positive impact on soil microbial diversity, the majority of studies observed no significant effect of rotations on soil bacterial diversity, including those with or without legume crops (Venter et al., 2016). Despite the lack of changes in α-diversity, we did observe compositional shifts of certain bacterial phyla in the bulk soil in response to pulse frequency. We sampled during the final year and second cycle of the 4 years rotations (i.e., 8th year of the field trial) when all plots were seeded to wheat so these shifts are likely a building legacy effect, particularly for the Bacteriodetes, which exhibited the strongest shift in response to pulse frequency. These shifts may also be related to the increased nitrogen fixation potential and higher residual soil nitrogen associated with higher pulse frequency in the crop rotations (Niu et al., 2017; Clúa et al., 2018; Liu J. et al., 2020).

The fungal community was more sensitive to pulse frequency than the bacterial community in our study, particularly the Ascomycota, which appear to be a dominant node in the soil microbial community network. We also found that putative fungal plant pathogens, which primarily belonged to the Ascomycota, significantly increased in rotations with higher pulse frequencies. Different crops can accumulate and select specific soil microbial taxa in the rhizosphere and bulk soil, which can be linked to either beneficial microbes that form symbiotic relationships with host plants and/or promote plant growth, or pathogens that infect specific host plant species (Zak et al., 2003). The network analysis also showed an increased abundance of dominant (nodes) Ascomycota taxa (including fungal pathotrophs and plant pathogens) that had more competitive interactions with Acidobacteria, Actinobacteria, Proteobacteria, and Planctomycetes in rotations with high pulse frequency. Previous studies have reported that certain pathogens can modify their host plant associated microbial community by building up syntrophic interactions with certain bacteria phyla, which can either directly help pathogen growth or inhibit certain bacterial phyla that have antagonistic influences on pathogens (Snelders et al., 2020; Wen et al., 2020). The competitive interactions between Ascomycota and other soil microbial phyla we identified in our study could be due to selective effects of potential pathogens on the soil microbial community. Overall, our results align with previous studies that showed that including two or more phases of pulse crops in 4 years rotations increases the proportion of fungal plant pathogens in soil, particularly those that belong to the Ascomycota such as *Fusarium* sp. and *Alternaria* sp. (Bainard et al., 2017). The shift in community composition of the Ascomycetes and buildup of fungal plant pathogens was the strongest in the bulk versus the rhizosphere soil, which likely indicates a potential building legacy effect of pulse frequency in this long term rotation experiment.

The network analysis revealed that pulse frequency altered the stability of the soil microbial communities. We found a higher ratio of identified edges to nodes and shorter average path distance under high pulse frequency rotations than low pulse frequency rotations in both the rhizosphere and bulk soil. This indicated that including more pulse crops in rotations could create favorable niches to select specific microbial taxa that can build up more intensive interactions within the community (Shi et al., 2016; Jiang et al., 2017). However, we found more negative interactions, shorter average path distance, lower average clustering coefficient and higher betweenness centrality numbers in the rhizosphere and bulk soil microbial communities under high pulse frequency rotations. This suggests there are more competitive interactions within soil microbial communities under rotations with high pulse frequency than their corresponding networks under rotations with low pulse frequency. This may lead to relatively unstable microbial networks as these increased interactions could be more easily interfered by other factors such as changes in environmental conditions (He et al., 2017).

We also found that higher pulse frequency changed the diversity and composition of keystone taxa of the soil microbial communities. Keystone taxa are highly related to soil microbial community structure and function, especially when these taxa are connectors in the community (Herren and McMahon, 2018). Based on previous studies, keystone taxa can alter the soil microbial community by modifying biotic connectivity within the community and changing the response of the microbial community to abiotic factors and their symbiotic relationships with host plants (Berry and Widder, 2014; Agler et al., 2016; Banerjee et al., 2018; Herren and McMahon, 2018). This can result in changes to the biological and ecological functions of the soil microbial community (Wittwer et al., 2017; Toju et al., 2018; Banerjee et al., 2019). In our study, we found that keystone taxa that functioned as connectors within the rhizosphere and bulk soil microbial communities increased under rotations with high pulse frequency. This may be an indicator that pulse frequency in crop rotations may be a contributing factor to causing functional shifts in the soil microbial community. A high proportion of keystone taxa altered by pulse frequency belonged to Acidobacteria, Proteobacteria, and Actinobacteria, which have been found to play critical biological and ecological functions in various ecosystems, including agroecosystems (Banerjee et al., 2016, 2018). Furthermore, some rare microbial taxa belonging to Bacteroidetes, Cyanobacteria, and Armatimonadetes were identified as keystone taxa (either module hubs or connectors), especially under rotations with high pulse frequency. These rare taxa have recently been reported as critical rare soil microbial species that can be major drivers of ecosystem multifunctionality in long-term agroecosystems (Chen et al., 2020). As a result, changes to these keystone taxa that belong to these bacterial phyla in our study due to high pulse frequency could reflect potential biological and ecological function changes to the soil microbial community.

In rhizosphere soil, we identified pH and EC as the primary environmental factors that influenced the microbial community, which has been commonly reported by other studies (Adviento-Borbe et al., 2006; Geisseler and Scow, 2014; Ratzke et al., 2020). In our study, the effect of pH and EC varied depending on pulse frequency, as modules containing the keystone taxa were more sensitive to pH and EC under low to medium pulse frequency. However, increased pulse frequency did not significantly alter the soil pH and EC in rhizosphere soils. This suggests that pulse frequency may influence the abundance of microbial taxa that are more responsive to pH and EC. Similarly, although bulk soil potassium didn’t significantly change with increased pulse frequency, potassium showed more negative interactions on the soil microbial community under low pulse frequency, but more positive interactions with microbes under higher pulse frequency. Overall, the various modules reacted to environmental factors differently, suggesting that microorganisms from these modules may be involved in different ecological functions.

Previous studies have reported that increased pulse phases in crop rotations has been shown to improve levels of soil available nitrogen (Niu et al., 2017), but we found that soil nitrate had stronger negative correlations with microbial modules in both rhizosphere and bulk soil, especially under high pulse frequency. Nitrogen is an essential nutrient for crop growth, but it is usually one of the strongest limiting factors in most agricultural systems worldwide (Stevens, 2019). However, the long-term accumulation of nitrogen inputs has been demonstrated to have inhibiting effects on soil microorganisms and to change the nutrient competition status of plants and microbes, symbiotic status of soil microbes on host plants, and alter C:N ratios, changing the growth rate of fungi and bacteria as they require different C:N ratios in soil (Zhang et al., 2018), which could explain some of the results observed in this study.

Interestingly, we found that soil sulfur showed a significant relationship with many identified microbial modules in this study. These relationships were generally more positive in rhizosphere soil and more negative in bulk soil, with similar results being shown in a study conducted on Luvisolic and Chernozemic soils in Canada (Lupwayi et al., 2001). Soil microorganisms play an important role in soil sulfur cycling. More than 95% of sulfur in soil exists in organic forms such as sulfate esters, sulphonates or amino acid sulfur which can be used by plants only after interconversion into inorganic sulfur by microorganisms (Kertesz and Mirleau, 2004), thus mineralization and immobilization of soil sulfur is largely dependent on the activities of soil microorganisms. Furthermore, sulfur is an essential element for some basic amino acids and enzymes to support the growth of microorganisms, and soil microbial sulfur is tightly related to soil total organic sulfur pools and soil carbon, nitrogen and phosphorus cycling (Banerjee and Chapman, 1996; Bünemann and Condron, 2007; Smolders et al., 2010). Sulfur is critical for legume crops to form nodules and fix nitrogen, which has been documented for decades (Anderson and Spencer, 1949; Reckling et al., 2020). Considering sulfur deficiencies have been reported consistently in modern agriculture (McNeill et al., 2005) and different crop rotation designs can significantly affect soil sulfur (Mohammadi et al., 2011), rotations with different legume:cereal ratios could restructure soil microbial communities through host plant effects. Therefore, these interactions between sulfur and identified microbial modules identified in our study can be the result of either direct nutrient effects or indirect host plant effects, which requires further investigation.

Overall, we found that high pulse frequency in cropping systems can alter the composition and increase network interactions of soil microbial communities in both rhizosphere and bulk soil. The Ascomycetes and Bacteroidetes exhibited the strongest shift under rotations with higher pulse frequencies, particularly in the bulk soil, which may be an indicator of a building legacy effect following two cycles through the 4 years rotation. We also observed an increase in potential fungal pathogens and negative interactions in the soil microbial networks under high pulse frequency, highlighting the risks associated with incorporating pulse crops into crop rotations.

## Data Availability Statement

The datasets presented in this study can be found in online repositories. The names of the repository/repositories and accession number(s) can be found below: NCBI BioProject; PRJNA694691, PRJNA694475, PRJNA694839, PRJNA694704, PRJNA717779, and PRJNA694448.

## Author Contributions

LB and BE designed and were responsible for conducting the field and lab work. TY and LB conducted the data analysis and wrote the manuscript. All authors contributed to editing the manuscript and approved the final version.

## Conflict of Interest

The authors declare that the research was conducted in the absence of any commercial or financial relationships that could be construed as a potential conflict of interest.

## References

[B1] Adviento-Borbe M. A. Doran J. W. Drijber R. A. Dobermann A. (2006). Soil electrical conductivity and water content affect nitrous oxide and carbon dioxide emissions in intensively managed soils. *J. Environ. Qual.* 35 1999–2010. 10.2134/jeq2006.0109 17071868

[B2] Agler M. T. Ruhe J. Kroll S. Morhenn C. Kim S.-T. Weigel D. (2016). Microbial hub taxa link host and abiotic factors to plant microbiome variation. *PLoS Biol.* 14:e1002352. 10.1371/journal.pbio.1002352 26788878 PMC4720289

[B3] Anderson A. J. Spencer D. (1949). Molybdenum and sulphur in symbiotic nitrogen fixation. *Nature* 164 273–274. 10.1038/164273a0 18139365

[B4] Bainard L. Evans B. Malis E. Yang T. Bainard J. (2020). Influence of annual plant diversity on forage productivity and nutrition, soil chemistry, and soil microbial communities. *Front. Sustain. Food Syst.* 4:560479. 10.3389/fsufs.2020.560479

[B5] Bainard L. D. Navarro-Borrell A. Hamel C. Braun K. Hanson K. Gan Y. (2017). Increasing the frequency of pulses in crop rotations reduces soil fungal diversity and increases the proportion of fungal pathotrophs in a semiarid agroecosystem. *Agric. Ecosyst. Environ.* 240 206–214. 10.1016/j.agee.2017.02.020

[B6] Banerjee M. R. Chapman S. J. (1996). The significance of microbial biomass sulphur in soil. *Biol. Fert. Soils* 22 116–125. 10.1007/BF00384442

[B7] Banerjee S. Kirkby C. A. Schmutter D. Bissett A. Kirkegaard J. A. Richardson A. E. (2016). Network analysis reveals functional redundancy and keystone taxa amongst bacterial and fungal communities during organic matter decomposition in an arable soil. *Soil Biol. Biochem.* 97 188–198. 10.1016/j.soilbio.2016.03.017

[B8] Banerjee S. Schlaeppi K. Van Der Heijden M. G. (2018). Keystone taxa as drivers of microbiome structure and functioning. *Nat. Rev. Microbiol.* 16 567–576. 10.1038/s41579-018-0024-1 29789680

[B9] Banerjee S. Walder F. Büchi L. Meyer M. Held A. Y. Gattinger A. (2019). Agricultural intensification reduces microbial network complexity and the abundance of keystone taxa in roots. *ISME J.* 13 1722–1736. 10.1038/s41396-019-0383-2 30850707 PMC6591126

[B10] Barbieri P. Pellerin S. Nesme T. (2017). Comparing crop rotations between organic and conventional farming. *Sci. Rep.* 7:13761. 29062017 10.1038/s41598-017-14271-6 PMC5653822

[B11] Berry D. Widder S. (2014). Deciphering microbial interactions and detecting keystone species with co-occurrence networks. *Front. Microbiol.* 5:219. 10.3389/fmicb.2014.00219 24904535 PMC4033041

[B12] Bünemann E. K. Condron L. M. (2007). “Phosphorus and sulphur cycling in terrestrial ecosystems,” in *Nutrient Cycling in Terrestrial Ecosystems*, eds Marschner P. Rengel Z. (Berlin: Springer), 65–92. 10.1007/978-3-540-68027-7_3

[B13] Caporaso J. G. Lauber C. L. Walters W. A. Berg-Lyons D. Huntley J. Fierer N. (2012). Ultra-high-throughput microbial community analysis on the illumina HiSeq and MiSeq platforms. *ISME J*. 6 1621–1624. 10.1038/ismej.2012.8 22402401 PMC3400413

[B14] Chen Q.-L. Ding J. Zhu D. Hu H.-W. Delgado-Baquerizo M. Ma Y.-B. (2020). Rare microbial taxa as the major drivers of ecosystem multifunctionality in long-term fertilized soils. *Soil Biol. Biochem.* 141:107686. 10.1016/j.soilbio.2019.107686

[B15] Clúa J. Roda C. Zanetti M. E. Blanco F. A. (2018). Compatibility between legumes and rhizobia for the establishment of a successful nitrogen-fixing symbiosis. *Genes* 9:125. 10.3390/genes9030125 29495432 PMC5867846

[B16] Delavaux C. S. Bever J. D. Karppinen E. M. Bainard L. D. (2020). Keeping it cool: soil sample cold pack storage and DNA shipment up to 1 month does not impact metabarcoding results. *Ecol. Evol*. 10 4652–4664. 10.1002/ece3.6219 32551050 PMC7297747

[B17] Deng Y. Jiang Y. H. Yang Y. He Z. Luo F. Zhou J. (2012). Molecular ecological network analyses. *BMC Bioinformatics* 13:113. 10.1186/1471-2105-13-113 22646978 PMC3428680

[B18] Drinkwater L. E. Wagoner P. Sarrantonio M. (1998). Legume-based cropping systems have reduced carbon and nitrogen losses. *Nature* 396 262–265. 10.1038/24376

[B19] Edgar R. C. (2013). UPARSE: highly accurate OTU sequences from microbial amplicon reads. *Nat. Methods* 10 996–998. 10.1038/nmeth.2604 23955772

[B20] Foyer C. H. Lam H. M. Nguyen H. T. Siddique K. H. Varshney R. K. Colmer T. D. (2016). Neglecting legumes has compromised human health and sustainable food production. *Nat. Plants* 2:16112. 10.1038/nplants.2016.112 28221372

[B21] Gan Y. Hamel C. O’donovan J. T. Cutforth H. Zentner R. P. Campbell C. A. (2015). Diversifying crop rotations with pulses enhances system productivity. *Sci. Rep.* 5:14625. 10.1038/srep14625 26424172 PMC4589733

[B22] Geisseler D. Scow K. M. (2014). Long-term effects of mineral fertilizers on soil microorganisms – A review. *Soil Biol. Biochem.* 75 54–63. 10.1016/j.soilbio.2014.03.023

[B23] Gentry C. E. Willis R. B. (1988). Improved method for automated determination of ammonium in soil extracts. *Commun. Soil Sci. Plant Anal.* 19 721–737. 10.1080/00103628809367970

[B24] Guimerà R. Nunes Amaral L. A. (2005). Functional cartography of complex metabolic networks. *Nature* 433 895–900. 10.1038/nature03288 15729348 PMC2175124

[B25] Habekost M. Eisenhauer N. Scheu S. Steinbeiss S. Weigelt A. Gleixner G. (2008). Seasonal changes in the soil microbial community in a grassland plant diversity gradient four years after establishment. *Soil Biol. Biochem.* 40 2588–2595. 10.1016/j.soilbio.2008.06.019

[B26] Hamel C. Gan Y. Sokolski S. Bainard L. D. (2018). High frequency cropping of pulses modifies soil nitrogen level and the rhizosphere bacterial microbiome in 4-year rotation systems of the semiarid prairie. *Appl. Soil Ecol.* 126 47–56. 10.1016/j.apsoil.2018.01.003

[B27] Harm J. Bettany J. Halstead E. (1973). A soil test for sulphur and interpretative criteria for Saskatchewan. *Commun. Soil Sci. Plant Anal.* 4 219–231. 10.1080/00103627309366440

[B28] He D. Shen W. Eberwein J. Zhao Q. Ren L. Wu Q. L. (2017). Diversity and co-occurrence network of soil fungi are more responsive than those of bacteria to shifts in precipitation seasonality in a subtropical forest. *Soil Biol. Biochem.* 115 499–510. 10.1016/j.soilbio.2017.09.023

[B29] Herren C. M. McMahon K. D. (2018). Keystone taxa predict compositional change in microbial communities. *Environ. Microbiol.* 20 2207–2217. 10.1111/1462-2920.14257 29708645

[B30] Jensen E. S. Carlsson G. Hauggaard-Nielsen H. (2020). Intercropping of grain legumes and cereals improves the use of soil N resources and reduces the requirement for synthetic fertilizer N: a global-scale analysis. *Agron. Sustain. Dev.* 40:5. 10.1007/s13593-020-0607-x

[B31] Jiang Y. Li S. Li R. Zhang J. Liu Y. Lv L. (2017). Plant cultivars imprint the rhizosphere bacterial community composition and association networks. *Soil Biol. Biochem.* 109 145–155. 10.1016/j.soilbio.2017.02.010

[B32] Kertesz M. A. Mirleau P. (2004). The role of soil microbes in plant sulphur nutrition. *J. Exp. Bot.* 55 1939–1945. 10.1093/jxb/erh176 15181108

[B33] Khakbazan M. Gan Y. Bandara M. Huang J. (2020). Economics of pulse crop frequency and sequence in a wheat-based rotation. *J. Agron.* 112 2058–2080. 10.1002/agj2.20182

[B34] Kirkegaard J. Christen O. Krupinsky J. Layzell D. (2008). Break crop benefits in temperate wheat production. *Field Crops Res.* 107 185–195. 10.1016/j.fcr.2008.02.010

[B35] Kõljalg U. Nilsson R. H. Abarenkov K. Tedersoo L. Taylor A. F. S. Bahram M. (2013). Towards a unified paradigm for sequence-based identification of fungi. *Mol. Ecol*. 22 5271–5277. 10.1111/mec.12481 24112409

[B36] Lal R. (2017). “Chapter four – Improving soil health and human protein nutrition by pulses-based cropping systems,” in *Advances in Agronomy*, ed. Sparks D. L. (Cambridge, MA: Academic Press), 167–204. 10.1016/bs.agron.2017.05.003

[B37] Liu J. Yu X. Qin Q. Dinkins R. D. Zhu H. (2020). The impacts of domestication and breeding on nitrogen fixation symbiosis in legumes. *Front. Genet.* 11:00973. 10.3389/fgene.2020.00973 33014021 PMC7461779

[B38] Liu K. Bandara M. Hamel C. Knight J. D. Gan Y. (2020). Intensifying crop rotations with pulse crops enhances system productivity and soil organic carbon in semi-arid environments. *Field Crops Res.* 248 107657. 10.1016/j.fcr.2019.107657

[B39] Lupwayi N. Z. Monreal M. A. Clayton G. W. Grant C. A. Johnston A. M. Rice W. A. (2001). Soil microbial biomass and diversity respond to tillage and sulphur fertilizers. *Can. J. Soil Sci.* 81 577–589. 10.4141/S01-010

[B40] Martin K. J. Rygiewicz P. T. (2005). Fungal-specific PCR primers developed for analysis of the ITS region of environmental DNA extracts. *BMC Microbiol*. 5:28. 10.1186/1471-2180-5-28 15904497 PMC1156903

[B41] McNeill A. Eriksen J. Bergström L. Smith K. Marstorp H. Kirchmann H. (2005). Nitrogen and sulphur management: challenges for organic sources in temperate agricultural systems. *Soil Use Manag.* 21 82–93. 10.1111/j.1475-2743.2005.tb00112.x

[B42] Miller J. J. Curtin D. (2006). “Electrical conductivity and soluble ions,” in *Soil Sampling and Methods of Analysis*, 2 Edn, eds Carter M. R. Gregorich E. G. (Boca Raton, FL: CRC Press).

[B43] Mohammadi K. Ghalavand A. Aghaalikhani M. Heidari G. Shahmoradi B. Sohrabi Y. (2011). Effect of different methods of crop rotation and fertilization on canola traits and soil microbial activity. *Aust. J. Crop Sci.* 5:1261.

[B44] Navarro-Borrell A. Shi Y. Gan Y. Bainard L. D. Germida J. J. Hamel C. (2017). Fungal diversity associated with pulses and its influence on the subsequent wheat crop in the Canadian prairies. *Plant Soil* 414 13–31. 10.1007/s11104-016-3075-y

[B45] Nguyen N. H. Song Z. Bates S. T. Branco S. Tedersoo L. Menke J. (2016). FUNGuild: an open annotation tool for parsing fungal community datasets by ecological guild. *Fungal Ecol.* 20 241–248. 10.1016/j.funeco.2015.06.006

[B46] Niu Y. Bainard L. D. Bandara M. Hamel C. Gan Y. (2017). Soil residual water and nutrients explain about 30% of the rotational effect in 4-yr pulse-intensified rotation systems. *Can. J. Plant Sci.* 97 852–864. 10.1139/cjps-2016-0282

[B47] Olesen J. M. Bascompte J. Dupont Y. L. Jordano P. (2007). The modularity of pollination networks. *Proc. Natl. Acad. Sci. U.S.A.* 104 19891–19896. 10.1073/pnas.0706375104 18056808 PMC2148393

[B48] Ratzke C. Barrere J. Gore J. (2020). Strength of species interactions determines biodiversity and stability in microbial communities. *Nat. Ecol. Evol*. 4 376–383. 10.1038/s41559-020-1099-4 32042124

[B49] Reckling M. Bergkvist G. Watson C. A. Stoddard F. L. Bachinger J. (2020). Re-designing organic grain legume cropping systems using systems agronomy. *Eur. J. Agron.* 112:125951. 10.1016/j.eja.2019.125951

[B50] Schönhart M. Schmid E. Schneider U. A. (2011). CropRota – A crop rotation model to support integrated land use assessments. *Eur. J. Agron.* 34 263–277. 10.1016/j.eja.2011.02.004

[B51] Schumacher B. A. (2002). *Methods for the Determination of Total Organic Carbon (TOC) in Soils and Sediments.* Washington, DC: United States Environmental Protection Agency, 1–24.

[B52] Shi S. Nuccio E. E. Shi Z. J. He Z. Zhou J. Firestone M. K. (2016). The interconnected rhizosphere: high network complexity dominates rhizosphere assemblages. *Ecol. Lett.* 19 926–936. 10.1111/ele.12630 27264635

[B53] Smolders A. J. P. Lucassen E. C. H. E. T. Bobbink R. Roelofs J. G. M. Lamers L. P. M. (2010). How nitrate leaching from agricultural lands provokes phosphate eutrophication in groundwater fed wetlands: the sulphur bridge. *Biogeochemistry* 98 1–7. 10.1007/s10533-009-9387-8

[B54] Snelders N. C. Rovenich H. Petti G. C. Rocafort M. Van Den Berg G. C. Vorholt J. A. (2020). Microbiome manipulation by a soil-borne fungal plant pathogen using effector proteins. *Nat. Plants* 6 1365–1374. 10.1038/s41477-020-00799-5 33139860

[B55] Stevens C. J. (2019). Nitrogen in the environment. *Science* 363 578–580. 10.1126/science.aav8215 30733401

[B56] Toju H. Tanabe A. S. Sato H. (2018). Network hubs in root-associated fungal metacommunities. *Microbiome* 6:116. 10.1186/s40168-018-0497-1 29935536 PMC6015470

[B57] Venter Z. S. Jacobs K. Hawkins H.-J. (2016). The impact of crop rotation on soil microbial diversity: a meta-analysis. *Pedobiologia* 59 215–223. 10.1016/j.pedobi.2016.04.001

[B58] Wang Q. Garrity G. M. Tiedje J. M. Cole J. R. (2007). Naive bayesian classifier for rapid assignment of rRNA sequences into the new bacterial taxonomy. *Appl. Environ. Microbiol* 73 5261–5267. 10.1128/AEM.00062-07 17586664 PMC1950982

[B59] Wen T. Zhao M. Liu T. Huang Q. Yuan J. Shen Q. (2020). High abundance of *Ralstonia solanacearum* changed tomato rhizosphere microbiome and metabolome. *BMC Plant Biol.* 20:166. 10.1186/s12870-020-02365-9 32293273 PMC7160980

[B60] Willis A. Bunge J. (2015). Estimating diversity via frequency ratios. *Biom. Methodol*. 71 1042–1049. 10.1111/biom.12332 26038228

[B61] Wittwer R. A. Dorn B. Jossi W. Van Der Heijden M. G. (2017). Cover crops support ecological intensification of arable cropping systems. *Sci. Rep.* 7:41911. 10.1038/srep41911 28157197 PMC5291223

[B62] Zak D. R. Holmes W. E. White D. C. Peacock A. D. Tilman D. (2003). Plant diversity, soil microbial communities, and ecosystem function: are there any links? *Ecology* 84 2042–2050. 10.1890/02-0433

[B63] Zhang T. A. Chen H. Y. H. Ruan H. (2018). Global negative effects of nitrogen deposition on soil microbes. *ISME J.* 12 1817–1825. 10.1038/s41396-018-0096-y 29588494 PMC6018792

[B64] Zhao J. Yang Y. Zhang K. Jeong J. Zeng Z. Zang H. (2020). Does crop rotation yield more in China? A meta-analysis. *Field Crops Res.* 245:107659. 10.1016/j.fcr.2019.107659

