# Supplementary material for: Pulse Frequency in Crop Rotations Alters Soil Microbial Community Networks and the Relative Abundance of Fungal Plant Pathogens
Source: Front Microbiol. 2021 May 26;12:667394. doi: 10.3389/fmicb.2021.667394 (PMC8189174; doi:10.3389/fmicb.2021.667394)

**Supplementary Material**

Pulse frequency in crop rotations alters soil microbial community networks and the relative abundance of fungal plant pathogens

Tony Yang^1^, Bianca Evans^1^, Luke D. Bainard^2^*

^1^Swift Current Research and Development Centre, Agriculture and Agri-Food Canada, Swift Current, SK, Canada

^2^Agassiz Research and Development Centre, Agriculture and Agri-Food Canada, Agassiz, BC, Canada

*Corresponding author: luke.bainard@canada.ca

Table S1. Wheat growth under different legume frequency. Means with different letters from the same column are significantly different (*p* < 0.05).

| Pulse frequency | tiller (m^2^) | biomass (g/m^2^) | height (cm) | seeds per plant | yield  (kg/ha) |
| --- | --- | --- | --- | --- | --- |
| Low | 114±5b | 440.95±17.63b | 81.13±0.62 | 50±1.35 | 2239±78.5 |
| Medium | 128±4a | 515.81±19.85a | 81.96±1.18 | 54±3.36 | 2501±144.9 |
| High | 132±5a | 469.85±19.95b | 81.88±0.86 | 50±4.06 | 2338±40.62 |
| P-value | 0.021 | 0.031 | 0.778 | 0.476 | 0.56 |

Table S2. Influence of bulk and rhizosphere soil properties on the composition of the fungal and bacterial communities based on the PERMANOVA

|  | Rhizosphere soil | | |  | Bulk Soil | | |
| --- | --- | --- | --- | --- | --- | --- | --- |
| ***Fungi*** | F | R^2^ | *P* |  | F | R^2^ | *P* |
| S | 1.252 | 0.036 | 0.198 |  | 2.487 | 0.068 | <0.001 |
| K | 0.915 | 0.026 | 0.609 |  | 0.995 | 0.029 | 0.454 |
| N | 0.794 | 0.022 | 0.791 |  | 1.072 | 0.031 | 0.261 |
| P | 0.930 | 0.027 | 0.514 |  | 1.193 | 0.034 | 0.222 |
| OC | 1.443 | 0.041 | 0.040 |  | 1.052 | 0.030 | 0.346 |
| Total N | 0.825 | 0.024 | 0.747 |  | 0.930 | 0.027 | 0.546 |
| pH | 1.703 | 0.048 | 0.008 |  | 1.317 | 0.037 | 0.095 |
| EC | 1.086 | 0.031 | 0.248 |  | 1.341 | 0.038 | 0.081 |
| Moisture | 0.994 | 0.028 | 0.494 |  | 1.340 | 0.038 | 0.159 |
|  |  |  |  |  |  |  |  |
| ***Bacteria*** |  |  |  |  |  |  |  |
| S | 2.597 | 0.071 | 0.019 |  | 4.389 | 0.114 | 0.004 |
| K | 0.912 | 0.026 | 0.438 |  | 0.655 | 0.019 | 0.735 |
| N | 0.554 | 0.016 | 0.860 |  | 1.205 | 0.034 | 0.206 |
| P | 0.982 | 0.281 | 0.416 |  | 3.313 | 0.089 | 0.006 |
| OC | 1.354 | 0.038 | 0.152 |  | 0.803 | 0.023 | 0.572 |
| Total N | 1.134 | 0.032 | 0.354 |  | 1.106 | 0.032 | 0.296 |
| pH | 3.694 | 0.100 | 0.005 |  | 3.826 | 0.101 | 0.002 |
| EC | 2.567 | 0.070 | 0.046 |  | 4.208 | 0.110 | <0.001 |
| Moisture | 1.210 | 0.034 | 0.296 |  | 2.008 | 0.056 | 0.119 |


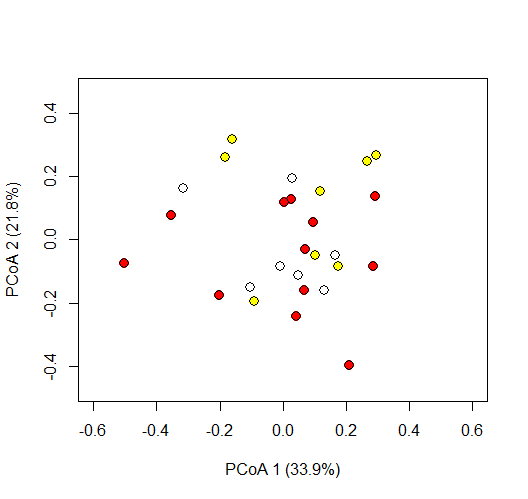


**E**


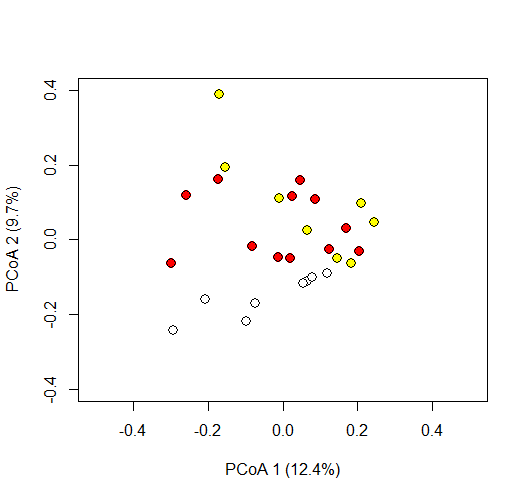


**C**


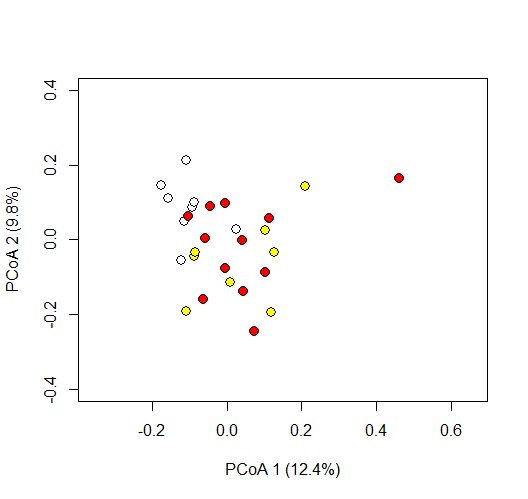


**A**


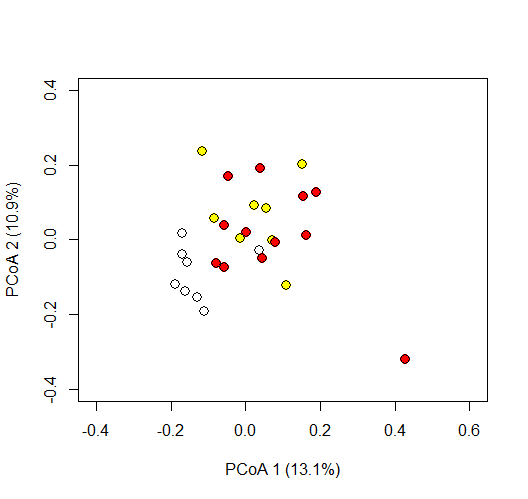


**B**


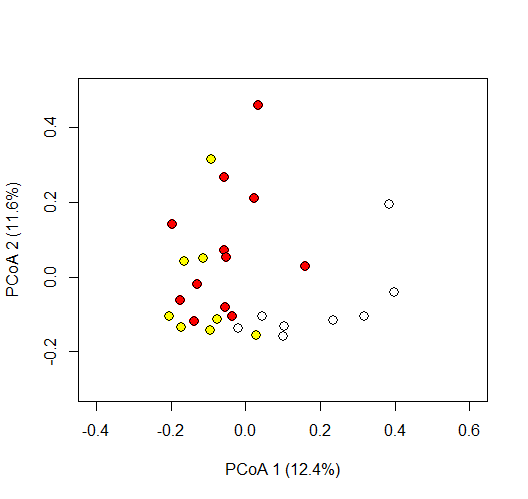


**D**


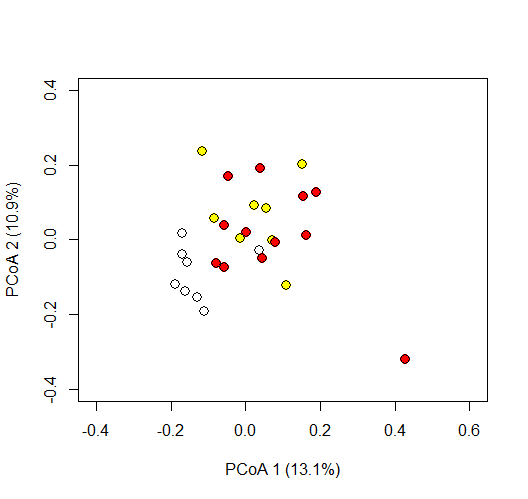


**B**

Fig. S1. Principal coordinates analysis (PCoA) of (A) rhizosphere fungal community, (B) rhizosphere Ascomycota community, (C) bulk soil fungal community, (D) bulk soil Ascomycota community, and (E) bulk soil Mortierellomycota community. The colour of the symbols corresponds to the level of pulse frequency (white = low, yellow = medium, red = high).


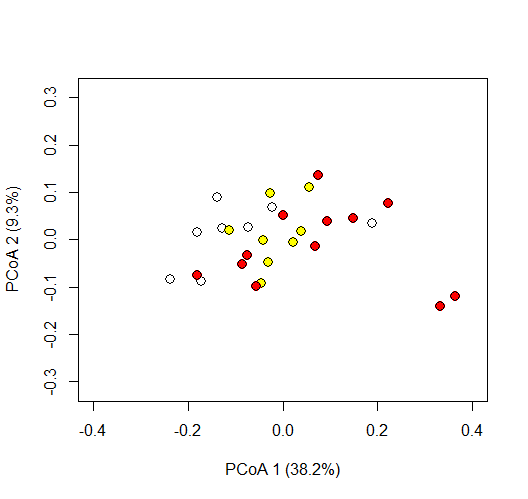

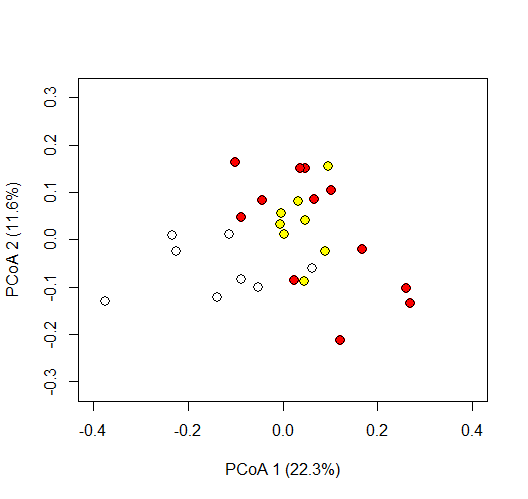

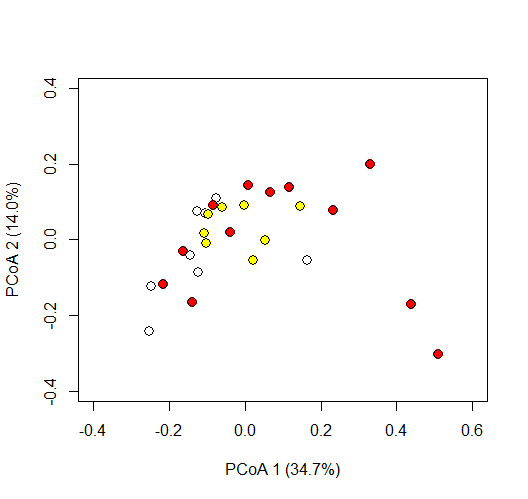

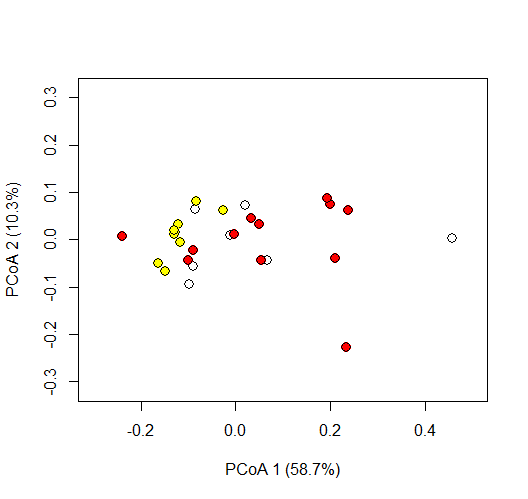

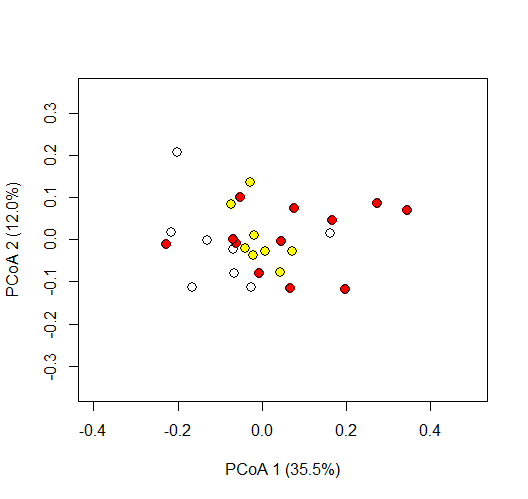

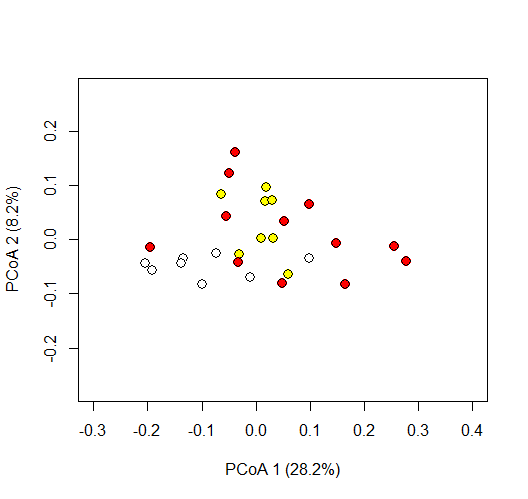


**A**

**B**

**C**

**D**

**E**

**F**

Fig. S2. Principal coordinates analysis (PCoA) of (A) rhizosphere soil Firmicutes community, (B) bulk soil Actinobacteria community, (C) bulk soil Armatimonadetes community, (D) bulk soil Bacteroidetes community, (E) bulk soil Gemmatimonadetes community, (F) bulk soil Proteobacteria community. The colour of the symbols corresponds to the level of pulse frequency (white = low, yellow = medium, red = high).


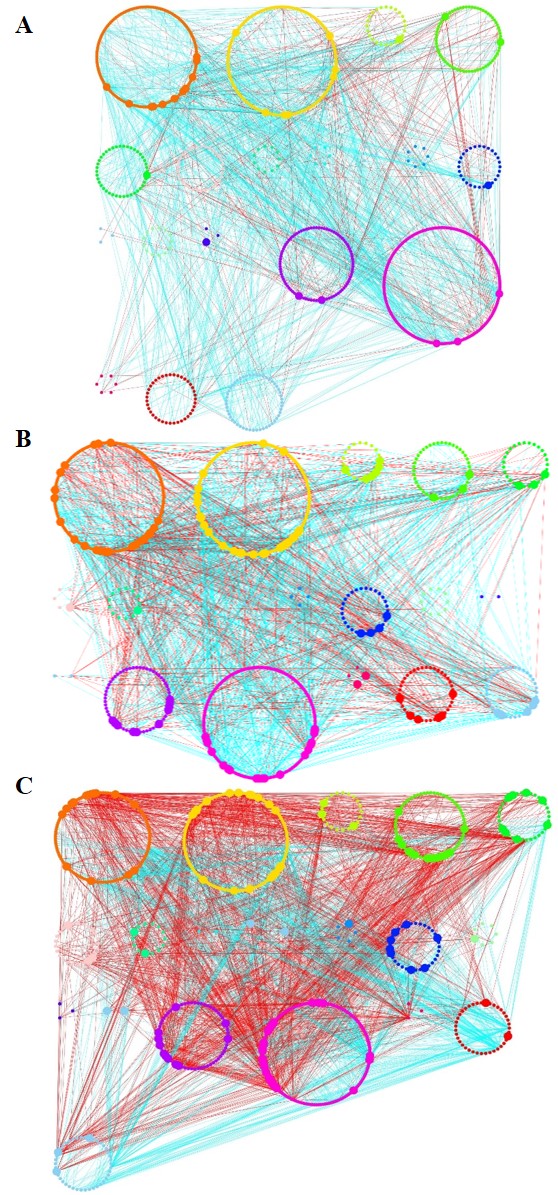


Fig. S3. Network analysis of A) low, B) medium and C) high pulse frequency influenced microbial community and dominant microbial OTUs (larger dots in network, OTUs with node degree larger than 10) in wheat rhizosphere soil at the phyla level. Blue lines between any connected two nodes indicates a positive relationship and red lines indicate a negative relationship.


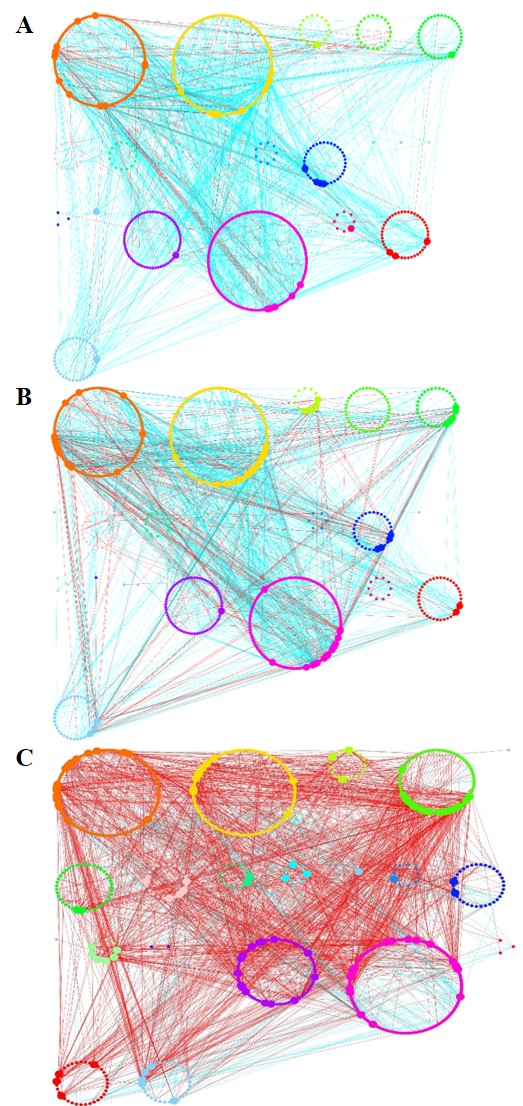


Fig. S4. Network analysis of A) low; B) medium and C) high pulse frequency influenced microbial community and dominant microbial OTUs (larger dots in network, OTUs with node degree larger than 10) in bulk soil at the phyla level. Blue lines between any connected two nodes indicates a positive relationship and red lines indicate a negative relationship.

Fig. S5. Module-EigenGene analysis that determined how the soil microbial modules interacted with environmental parameters. Modules with thick black frames are modules that include most keystone OTUs. The red color indicates a positive correlation and green color indicates a negative correlation. The number in each cell represents the correlation coefficient along with the corresponding *P* value in brackets. A) low pulse frequency in rhizosphere soil; B) medium pulse frequency in rhizosphere soil; C) high pulse frequency in rhizosphere soil; D) low pulse frequency in bulk soil; E) medium pulse frequency in bulk soil; F) high pulse frequency in bulk soil.

(A)


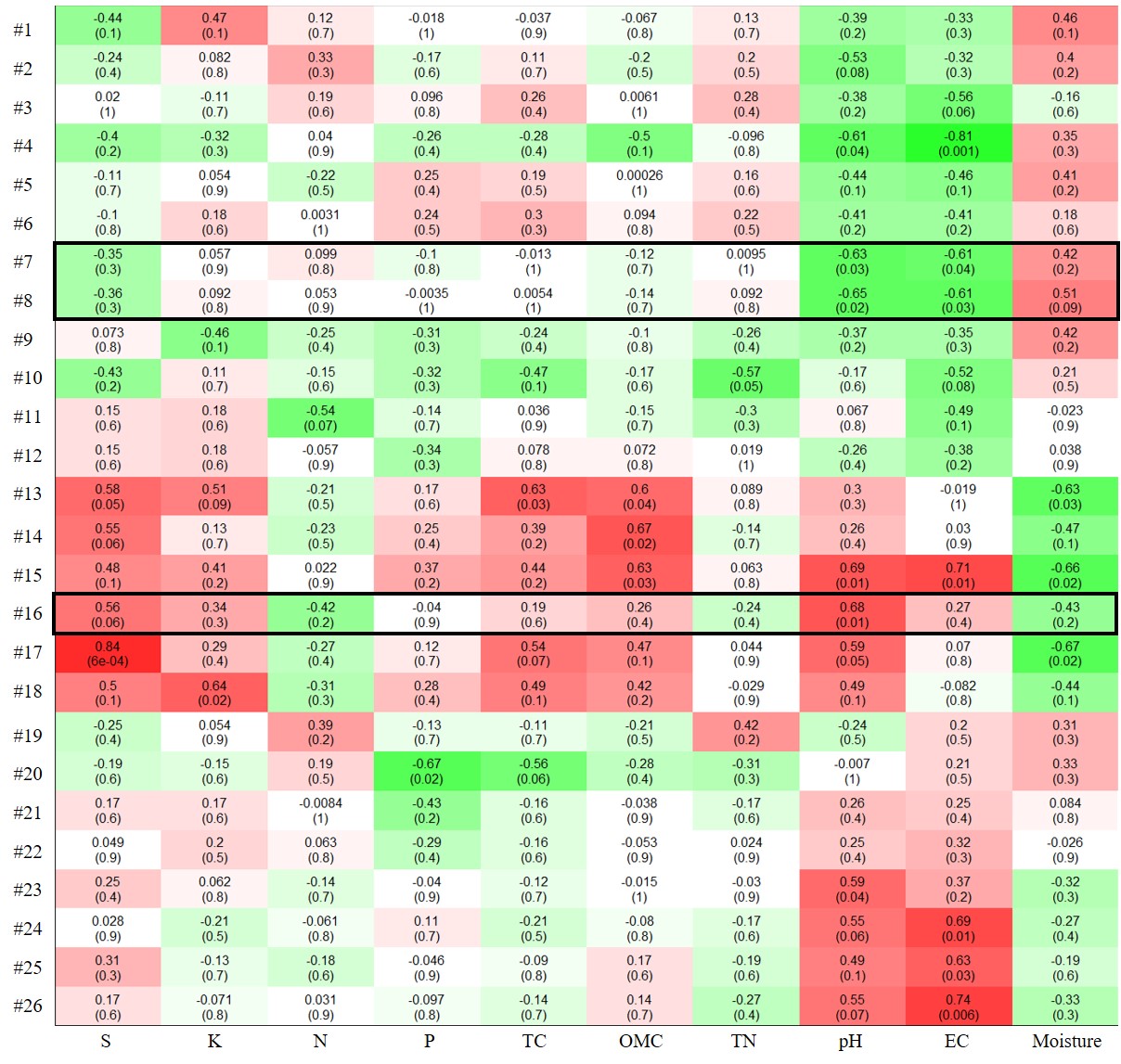


(B)


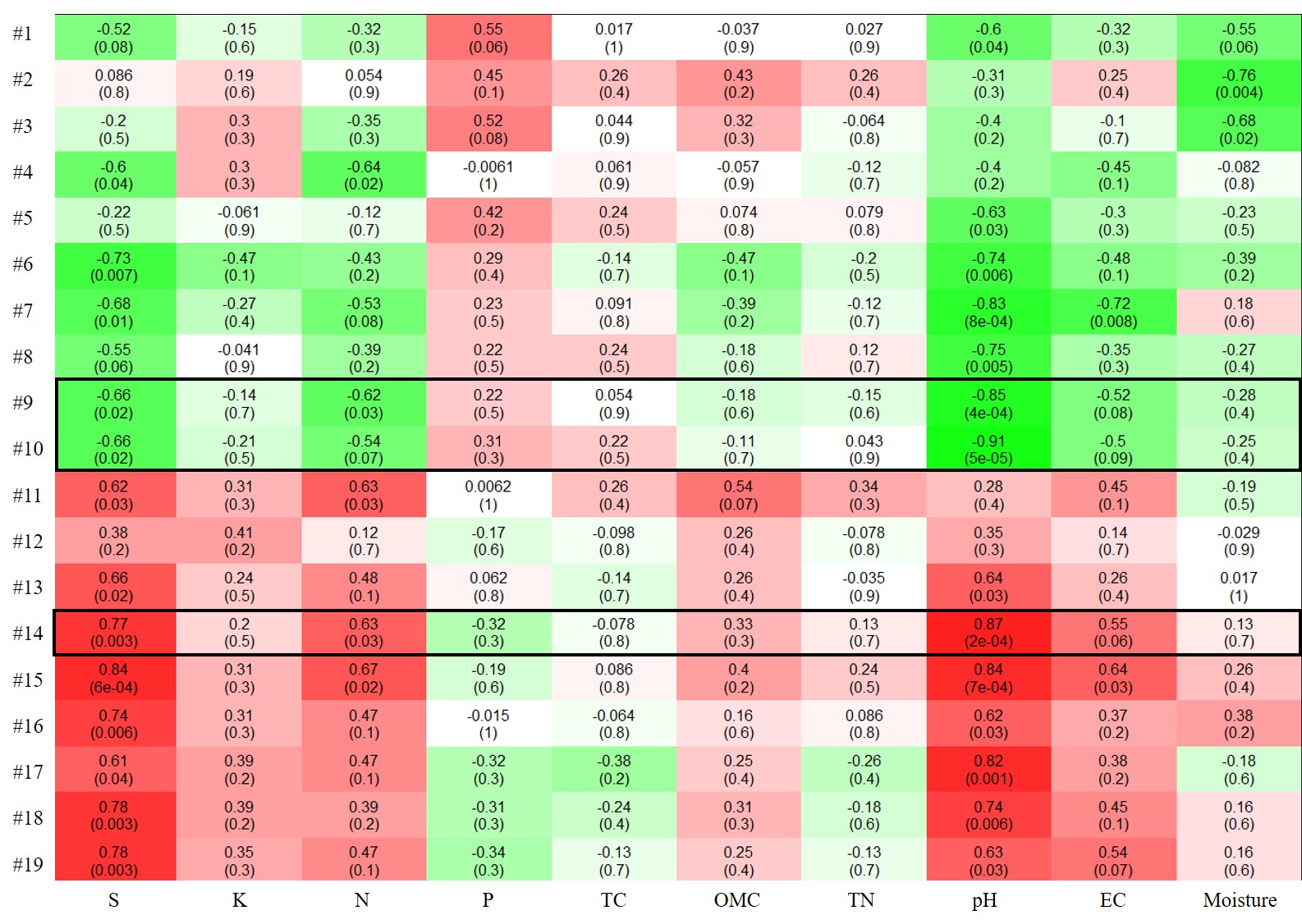


(C)


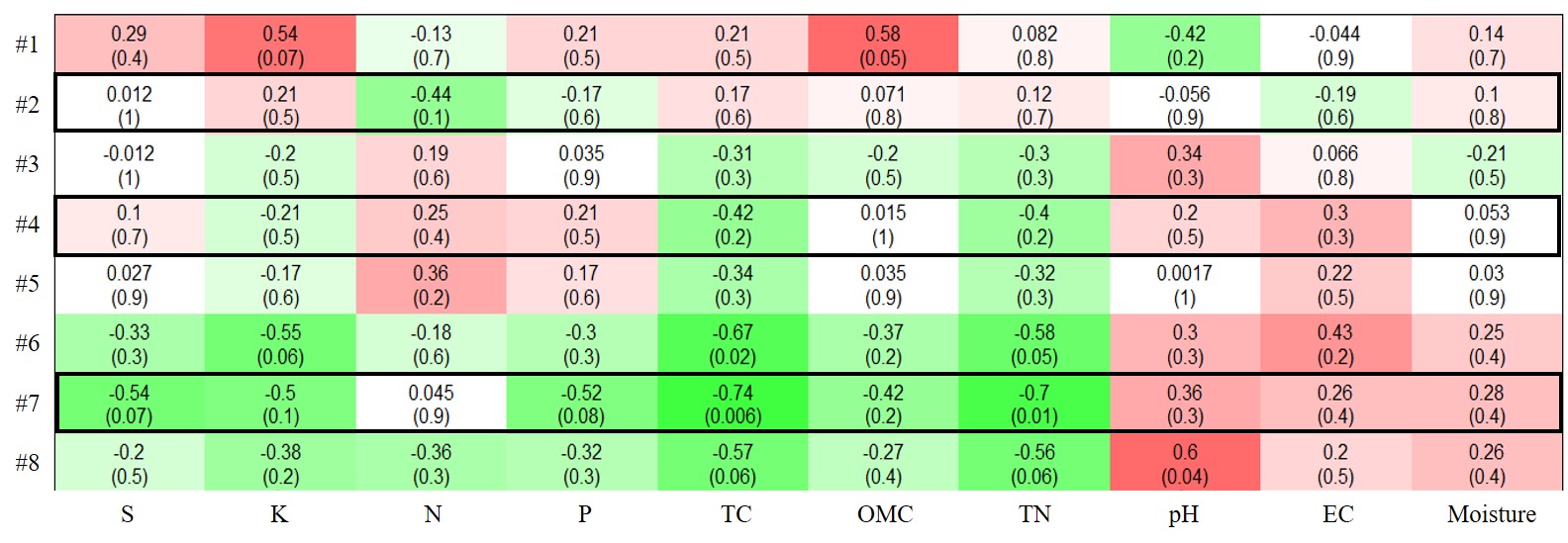


(D)


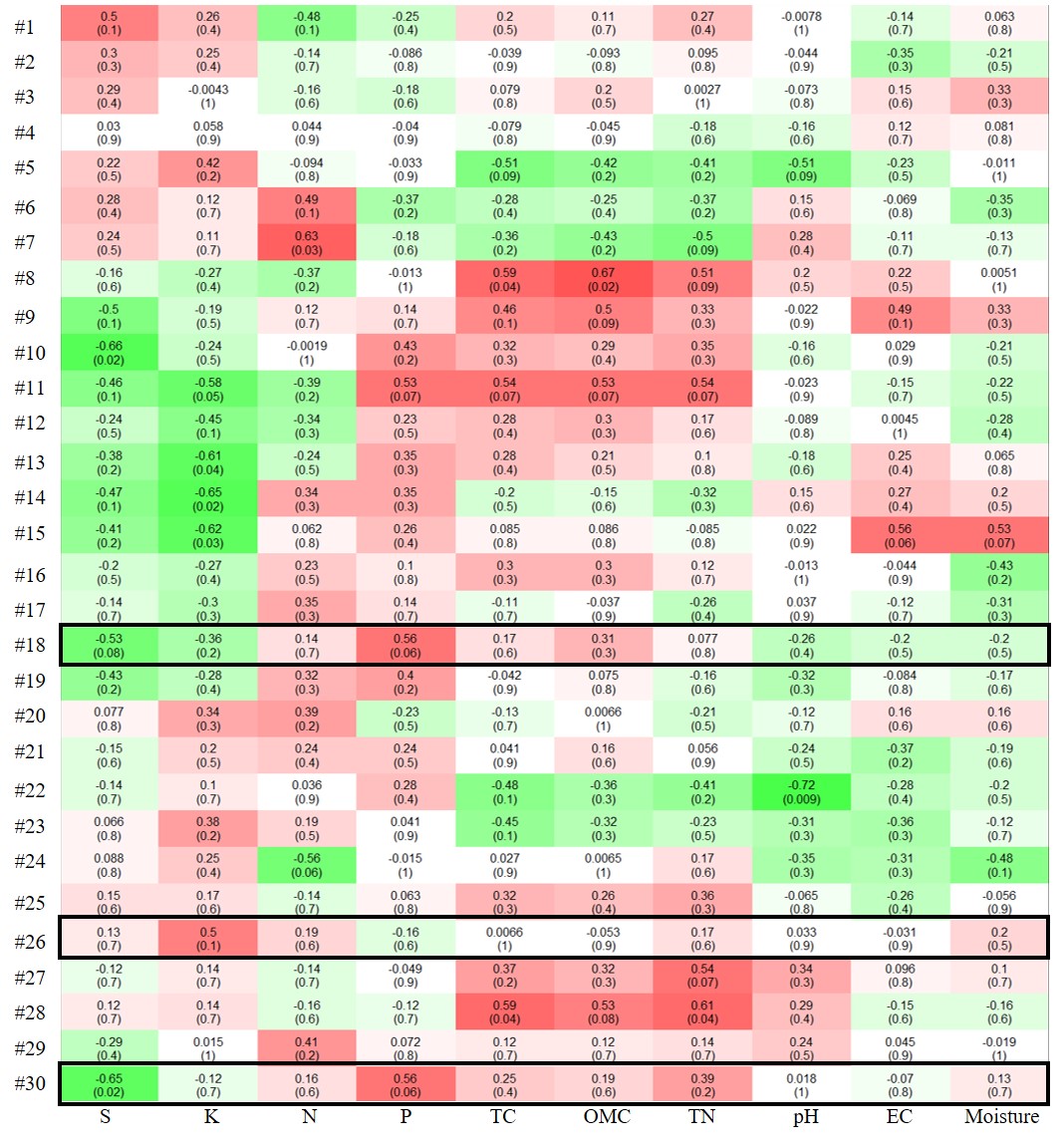


(E)


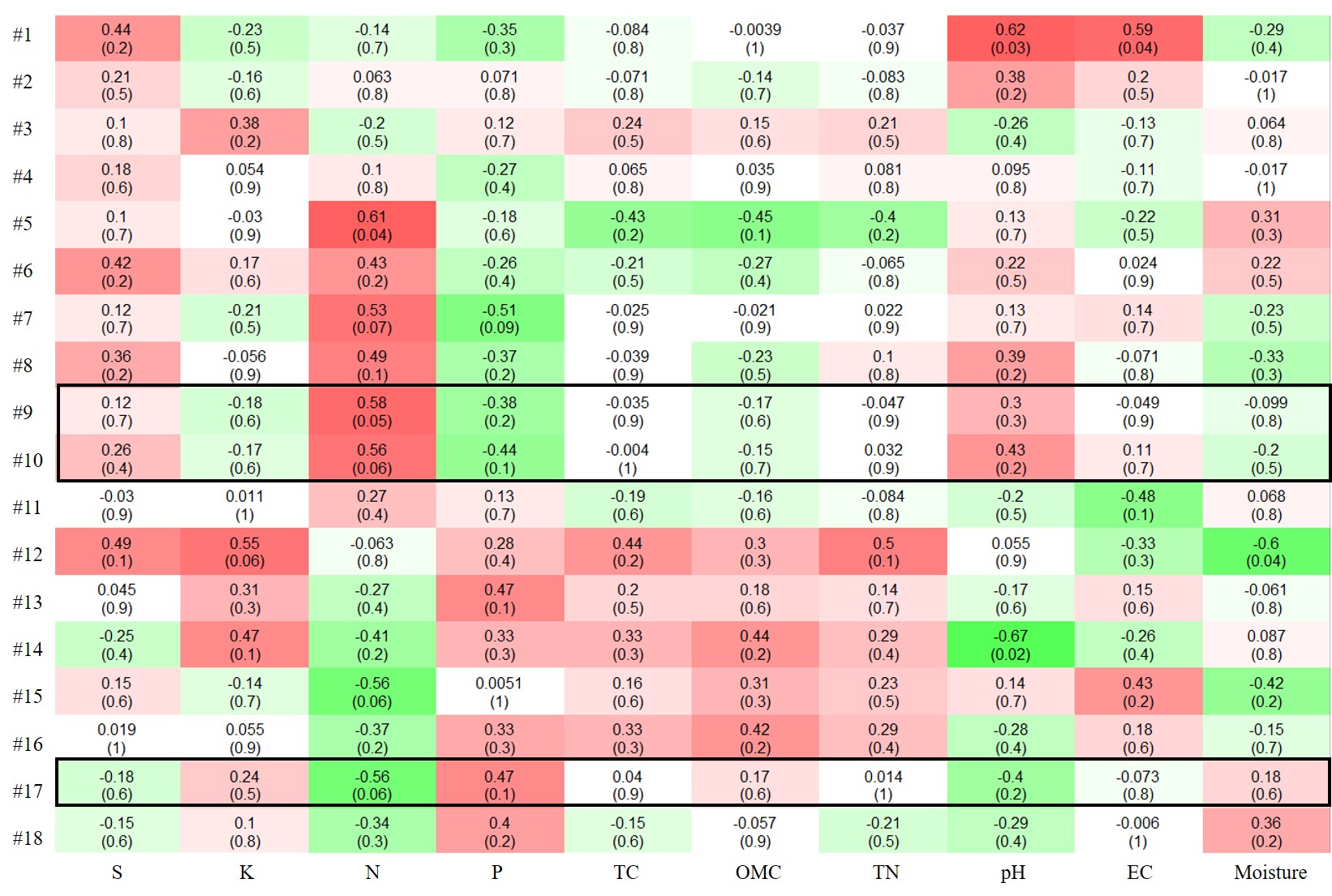


(F)


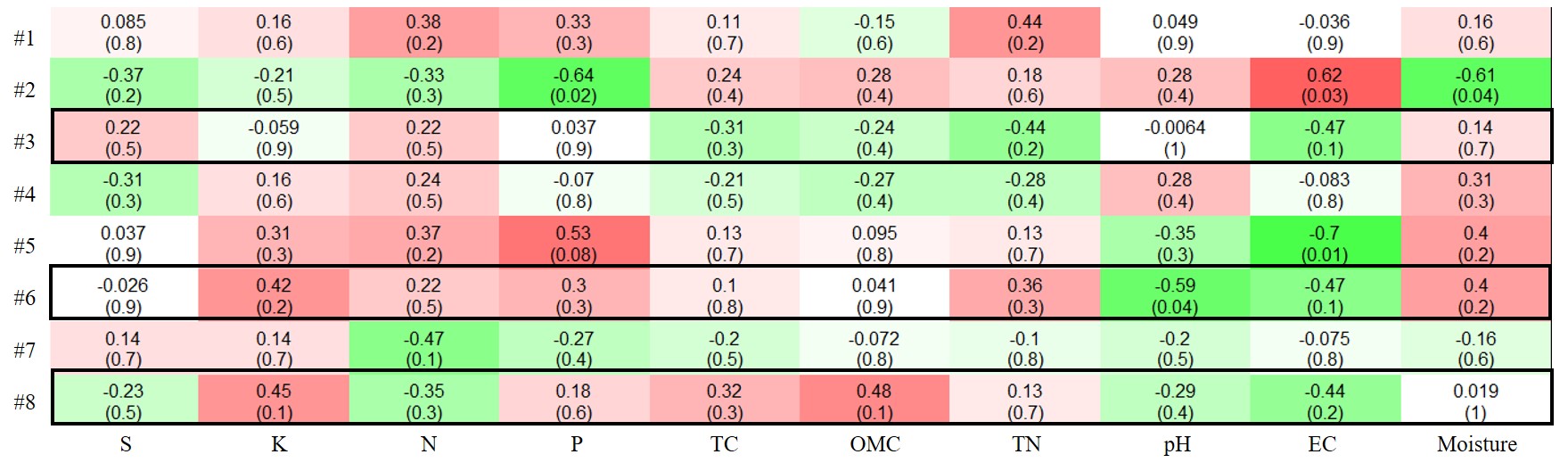

Supplement: Supplementary file 1 [file Table_1.DOCX]
